# The PAMINO-project: evaluating a primary care-based educational program to improve the quality of life of palliative patients

**DOI:** 10.1186/1472-684X-6-5

**Published:** 2007-05-29

**Authors:** Thomas Rosemann, Katja Hermann, Antje Miksch, Peter Engeser, Joachim Szecsenyi

**Affiliations:** 1 Department of General Practice and Health Services Research, University Hospital of Heidelberg, Vosstr. 2, 69115 Heidelberg, Germany

## Abstract

**Background:**

The care of palliative patients challenges the health care system in both quantity and quality. Especially the role of primary care givers needs to be strengthened to provide them with the knowledge and the confidence of applying an appropriate end-of-life care to palliative patients. To improve health care services for palliative patients in primary care, interested physicians in and around Heidelberg, Germany, are enabled to participate in the community-based program 'Palliative Medical Initiative North Baden (PAMINO)' to improve their knowledge in dealing with palliative patients. The impact of this program on patients' health and quality of life remains to be evaluated.

**Methods/Design:**

The evaluation of PAMINO is a non-randomized, controlled study. Out of the group of primary care physicians who took part in the PAMINO program, a sample of 45 physicians and their palliative patients will be compared to a sample of palliative patients of 45 physicians who did not take part in the program. Every four weeks for 6 months or until death, patients, physicians, and the patients' family caregivers in both groups answer questions to therapy strategies, quality of life (QLQ-C15-PAL, POS), pain (VAS), and burden for family caregivers (BSFC). The inclusion of physicians and patients in the study starts in March 2007.

**Discussion:**

Although participating physicians value the increase in knowledge they receive from PAMINO, the effects on patients remain unclear. If the evaluation reveals a clear benefit for patients' quality of life, a larger-scale implementation of the program is considered.

**Trial registration**: The study was registered at ‘current controlled trials (CCT)’, registration number:   ISRCTN78021852.

## Background

For the health care system, palliative care is a challenge both in quantity and quality. Inpatient and outpatient care need to be improved. In Germany, each year more than 215.000 people die of the aftermath of malignant tumours [1,2], of which about 150.000 suffer unbearable pain. Regarding to a study of the German Hospice Foundation, in 2002 only 1.8% of dying people in Germany received structured palliative care and 4.3% were cared for in hospices [2]. To secure the highest possible quality in health care and to improve quality of life of dying patients, palliative care needs to be further established in the German health care system. Within this context, a network of well-educated primary care givers in an outpatient setting and the implementation of new community-based concepts of care within health care politics are of paramount importance.

As in other European countries, most patients (67 to 90%) wish to die at home [3-5], a mismatch to reality where the hospital is the most common site of death [5-7]. Many patients express fears of social isolation, loss of independence and self-reliance, and pain at the end of life. As a primary goal, outpatient care for the incurable should be managed at their home, which is often hampered by deficits in communication between patients and medical care givers. Patients prefer to die in the home setting they are used to, if they have positive experiences of specialist community-based palliative care services and primary care services [8,9]. Competent and supportive communication between patients and general practitioners, based on a trustful relationship, helps both patients and physicians to discuss treatment strategies and end-of-life care and reach mutual agreements. Half of the patients with malignant tumours would like to participate in their care on the foundation of such shared decision making. With general practitioners, who are long-time confidants of the patients in their role as family physicians, patients speak confidently about their wishes regarding end-of-life care. Thus, physicians are able to initiate palliative medical interventions in good time. Additionally, a strengthened primary care might meet the demands of patients for high-quality information and instructive face-to-face communication about their disease. After leaving hospital, many patients are not informed enough about the seriousness of their illness and the unfavourable prognosis; the involvement of the family physician might close this gap.

Although relatives and family caregivers face the same lack of information as the patients themselves, they play an important role for palliative care since 70–80% of patients in palliative situations highly esteem their relatives' opinion of decisions regarding their therapy [10,11]. A crucial task of general practitioners consists of perceiving the psychological and physical burden to the family caregivers and treating this challenging situation in an appropriate way, which meets the needs and abilities of both patients and caregivers. To avoid frustrations and insufficient information, family caregivers should be involved in and informed about the process of care at the end of life as soon and as continuously as possible. With the support from health care professionals, family caregivers are more satisfied with a home care [12], which is a major influence on their perceived physical and psychological burden. Therefore, the support of (care giving) relatives in the presence of psychological and physical strain and the prevention of a burn-out situation should receive great attention while building up palliative care in a primary care context. The family physician holds a principal position as the social relationship of a patient to doctors and caregivers is of major value to the dying. In connection with a holistic approach of primary care, the highest possible quality of life for the patients might be reached [13].

An important part of good palliative care is the best possible control of symptoms, especially the avoidance of unnecessary pain. The fear of severe pain has a far higher priority in the perception of patients than the fear of death. For the majority of patients, pain is the most distressing symptom. Still, pain is less often diagnosed than it is present and often treated inadequately [14,15]. Therefore, the training of general practitioners in pain therapy is of paramount importance for the build-up of outpatient palliative care. Furthermore, a community-based, holistic approach to care prevents unnecessary and expensive stays in hospital as well as insufficient treatments. It is essential to qualify general practitioners for this task through appropriate trainings to guarantee an outpatient care of high quality considering the demands and points of view of patients, family caregivers, and physicians alike.

Still, the care of palliative patients is a challenging and burdening task for general practitioners. Furthermore, it is associated with high financial expenses. The support and advice for the care of a qualified team of general practitioners and caregivers by the cooperation with a competent academic network may ease this often difficult situation for general practitioners. At the same time, this collaboration with a backing specialist and the offer of sufficient resources provides for the possibility to look after the patients at home even in critical situations.

Research in palliative medicine often confines to description; evaluations of outcome using standardized and valid tools as well as cost analyzes are rare. With the evaluation of a community-based training, we want to meet this deficit. If the evaluation shows a positive effect of the training, especially on the quality of life of palliative patients and on health economic aspects, it might positively influence a wider implementation of community-based palliative care led by general practitioners.

## Methods/Design

### Aim and design of the study

This study compares the outcomes between a multifaceted-based interdisciplinary training concept in palliative care in a primary care setting (PAlliative Medical Initiative NOrth Baden – PAMINO) and usual palliative care for patients with malignant tumours.

### Scientific hypotheses

Patients of general practitioners who participated in the educational courses of PAMINO have a higher quality of life at the end of their life than patients of general practitioners who did not participate in palliative care training.

The study is a (prospective) two-armed, controlled, non-randomized evaluation study.

### Sample size

Based on the primary outcome measure quality of life measured by the Palliative Care Outcome Scale (POS), we include 360 patients from 90 general practitioners. Each arm contains 180 patients of 45 general practitioners, assuming that every GP cares for 4 patients who are suitable and willing to participate in the study. Sample size was calculated with the Cluster Sample Size Calculator of the University of Aberdeen, assuming a standard deviation of .60 and a minimum difference of 2.0 (for individual items); power is set to 80% and level of significance to 5%.

### Recruitment of GPs

Until April 2007, about 90 primary care physicians in the German federal state of Baden-Wuerttemberg took part in the PAMINO educational courses and are therefore eligible to include patients for the intervention group. For the control group, general practitioners from the same region are selected whose office characteristics (rural or urban area) match those of the intervention group.

### Patient inclusion criteria

The GPs participating in the study include consecutively adult outpatients (at least 18 years of age) of whom they are the family physician. Patients need to be in a palliative situation with an oncological disease and an expected survival of up to 6 months, estimated by their physician. They have to give their informed and written consent to participate.

### Patient exclusion criteria

Patients with malignant tumours in a curative therapy situation or with an additional uncontrolled disease with a lower life expectancy than the tumour disease must not be included in the study. Insufficient German language skills also lead to the exclusion from participating in the study.

### Data collection

In order to compare responders with non-responders regarding sociodemographic variables, GPs create a participants' list with a link to the medical files.

At inclusion in the study, patients receive a questionnaire comprised of the Palliative Care Outcome Scale (POS), the Quality of Life Questionnaire (QLQ-C15-PAL), and a visual analogue scale for assessing pain. Patients are allowed to get help for filling out the questionnaire.

General practitioners collect data to diagnoses and therapies of the study patients. They also assess the patients' quality of life from the physicians' point of view using the staff version of the POS.

Patients are asked to appoint the relative or friend who primarily cares for them at home (apart from physicians and nurses). This caregiver receives the Burden Scale for Family Caregivers (BSFC) to assess the psychological weight of the palliative situation on primary caregivers.

Similar to the study procedure of Jordhoy et al. [9], these tools (for patients, physicians, and family caregivers) are administered monthly from enrolment to either death of the patient or the end of the 6-months observation period.

Process indicators (existence of patient will/advance directive, substitution arrangement in case of unavailability of the treating general practitioner, cooperation with nursing services) are assessed at the beginning of the observation period and at the end if changes occur.

### Outcome measures

As the primary outcome parameter, we observe the change of quality of life of patients in the intervention group (patients of general practitioners with PAMINO-training) compared to the control group (patients of general practitioners without PAMINO-training). Quality of life will be assessed by the German version of the Palliative Care Outcome Scale (POS [16]), and the Quality of Life Questionnaire Core-15 Palliative Care (QLQ-C15-PAL [17]) of the European Organisation for Research and Treatment of Cancer (EORTC). Table 1 summarizes the outcome measures used in this study.

**Table 1:** Outcome measures and instruments used in the study

**Outcome measures**	**Assessment instruments**	**Assessment times**	**Assessed from**
**Primary outcome measure**			
Quality of life	Quality of Life Questionnaire Core-15 Palliative Care (QLQ-C15-PAL)	Every four weeks for 6 months or until death	Patient
	Palliative Care Outcome Scale (POS) – Self rating		
	Palliative Care Outcome Scale (POS) – Staff rating		GP

**Secondary outcome measures**			
Pain	Visual Analogue Scale (VAS)	Every four weeks for 6 months or until death	Patient
Burden for family caregivers	Burden Scale for Family Caregivers (BSFC)		Family caregiver
Health service resource use	Questionnaire		GP
Therapy (drug-related and other)			GP
Concurrence of preferred and actual site of death		Study inclusion and end of study (6 months later)	GP
Documents (patient will, do-not-resuscitate order)			GP
Availability of family physician		Study inclusion	GP
Cooperation with nursing services			

The training will have an effect on the following secondary outcomes:

- a lower pain level as experienced by the patients and assessed by a visual analogue scale (VAS)

- lower burden for family caregivers as assessed by the Burden Scale for Family Caregivers (BSFC [18])

- less utilization of the health care system (primary and specialist care, nursing service) including emergency and hospital admittance

- in a higher proportion of patients the favoured and actual site of death concur

The effects of the training on the following process indicators are observed:

- drug therapy, especially for pain, in adherence to the guidelines of the WHO

- therapeutic elements of palliative medicine besides drug therapy

- the existence of documents such as advance directives, do-not-resuscitate orders, and health care proxy, treatment plan

- prescription of pain medication

- realization of substitution in case of unavailability of the treating family physician

- cooperation with nursing services

### Intervention

PAMINO contains a curriculum consisting of a qualifying training course, which is based on the training course of the German Medical Association (Bundesärztekammer) and the German Association for Palliative Medicine, as well as of subsequent network meetings and quality circles. The interdisciplinary training course is held at the University of Heidelberg and covers issues of psychology of pain, legal aspects, dialogs of clarification with patients, ethics and attitudes, pain therapy (in theory and case studies), symptom control and specialized pain therapy (including practical applications), dying and the requirements of dying people, communication and burn-out, palliation in geriatrics, and palliative care.

### Timeframe of the study

Invitations to participate in the study are mailed to the GPs in April 2007. In July, an information meeting with participating GPs is conducted after which the GPs start to include patients in the study. The observation period lasts 6 months.

### Description of risks

Serious risks or undesired effects of questionnaires have not been described in the literature. There are no specific risks related to the study.

### Ethical and legal aspects

#### Ethical principles

The study is being conducted in accordance with medical professional codex and the Helsinki Declaration as of 1996 as well as the German Federal Data Security Law (BDSG).

Study participation of patients is voluntary and can be cancelled at any time without provision of reasons and without negative consequences for their future medical care.

#### Patient informed consent

Previous to study participation patients receive written and oral information about the content and extent of the study, i.e. about potential benefits for their health and potential risks. They sign the informed consent form to accept.

#### Legal principles

Vote of the ethics committee

The study protocol was approved by the ethics committee of the University of Heidelberg in March 2007 (approval number 043/2007) with an unrestricted positive vote.

### Data security/disclosure of original documents

The patients' names and all other confidential information comply with medical confidentiality and are treated according to the German Federal Data Security Law (Bundesdatenschutzgesetz, BDSG). The results of the patient questionnaires are not accessible to the GPs. Questionnaires are directly mailed to the study centre by the patient.

All data and documents related to the study are stored on a protected central server of the University Hospital Heidelberg. Only direct members of the internal study team can access the respective files.

Intermediate and final reports are stored in the office of the Department of General Practice and Health Services Research at the University Hospital Heidelberg.

## Discussion

Although participating physicians value the increase in knowledge they receive from PAMINO, the effects on patients remain unclear. The program enables physicians to structured palliative care within a primary care setting, thus trying to improve out-patient care. A community-based and holistic approach should secure the highest possible quality of life for palliative patients. Their end-of-life wishes and concerns about pain and loss of self-reliance could be better met if general practitioners were trained accordingly. Patients should be able to receive adequate treatment for their pain symptoms from their GP, especially when s/he is backed by a specialist academic network. A GP-centred treatment and community-based approach should rather support longer independence and self-reliance. A longer-lasting relationship like the one between primary medical caregivers and patients enhances communication and thus shared-decision making. Communication is an important part of the curriculum as well as the support of family caregivers. The help they receive from their family member's GP should lead to less burn-out and less perceived burden on the caregivers' side, thus enabling them to be a better support for the patients as they are an important influence for the patient's decisions. The PAMINO program should therefore improve physicians' palliative care, patients' quality of life and family caregivers' perceived burden of care. If the evaluation reveals a clear benefit for patients' quality of life, a larger-scale implementation of the program is considered.

## Competing interests

The author(s) declare that they have no competing interests.

## Authors' contributions

TR is the principal investigator of the study, PE, AM and JS were involved in the planning of the study. KH and TR wrote the study protocol. All authors read and approved the final version of the manuscript.

## Pre-publication history

The pre-publication history for this paper can be accessed here:


